# Hypoxia induces calpain activity and degrades SMAD2 to attenuate TGFβ signaling in macrophages

**DOI:** 10.1186/s13578-015-0026-x

**Published:** 2015-07-04

**Authors:** Wei Cui, Jie Zhou, Nathalie Dehne, Bernhard Brüne

**Affiliations:** College of Life Sciences, Beijing Normal University, 100875 Beijing, China; Institute of Biochemistry I, Faculty of Medicine, Goethe-University Frankfurt, 60590 Frankfurt, Germany

**Keywords:** Hypoxia, TGFß, phospho-SMAD2, SMAD2 degradation, Calpain, Macrophages

## Abstract

**Background:**

Under inflammatory conditions or during tumor progression macrophages acquire distinct phenotypes, with factors of the microenvironment such as hypoxia and transforming growth factor β (TGFβ) shaping their functional plasticity. TGFβ is among the factors causing alternative macrophage activation, which contributes to tissue regeneration and thus, resolution of inflammation but may also provoke tumor progression. However, the signal crosstalk between TGFβ and hypoxia is ill defined.

**Results:**

Exposing human primary macrophages to TGFβ elicited a rapid SMAD2/SMAD3 phosphorylation. This early TGFβ-signaling remained unaffected by hypoxia. However, with prolonged exposure periods to TGFβ/hypoxia the expression of SMAD2 declined because of decreased protein stability. In parallel, hypoxia increased mRNA and protein amount of the calpain regulatory subunit, with the further notion that TGFβ/hypoxia elicited calpain activation. The dual specific proteasome/calpain inhibitor MG132 and the specific calpain inhibitor 1 rescued SMAD2 degradation, substantiating the ability of calpain to degrade SMAD2. Decreased SMAD2 expression reduced TGFβ transcriptional activity of its target genes thrombospondin 1, dystonin, and matrix metalloproteinase 2.

**Conclusions:**

Hypoxia interferes with TGFβ signaling in macrophages by calpain-mediated proteolysis of the central signaling component SMAD2.

**Electronic supplementary material:**

The online version of this article (doi:10.1186/s13578-015-0026-x) contains supplementary material, which is available to authorized users.

## Introduction

Macrophages are found throughout the body, where they contribute to tissue homeostasis and orchestrate innate as well as adaptive immune responses. They display a remarkable plasticity, which allows them to change their functional repertoire with regard to the environment they are facing. Numerous sub-phenotypes are described. Based on distinct functional roles and marker expression profiles an operative useful but over-simplifying nomenclature categorizes classical, i.e., pro-inflammatory and alternatively activated macrophages [1]. An alternatively activated macrophage phenotype is elicited by interleukin-4 (IL-4), IL-10, or transforming growth factor ß (TGFß), which are produced by helper 2 or regulatory T cells, tumor cells, or macrophages themselves during late stage immune responses and tumor cells. Monocyte-derived macrophages and tissue resident macrophages are found in tumors. These tumor-associated macrophages are known to support cancer progression rather than mounting anti-cancer responses. This tumor-supporting phenotype is elicited by cytokines like TGFß [2].

TGFß controls proliferation, differentiation, and fosters fibrosis. TGFß binds to transforming growth factor receptor 2 (TGFΒR2), which recruits and phosphorylates transforming growth factor receptor 1 (TGFΒR1), thereby activating downstream signaling [3]. SMAD proteins mediate canonical TGFß signaling, while the PI3K-AKT, MAPK pathway conveys non-canonical signals [4]. The canonical pathway phosphorylates regulatory SMADs via receptor activation. In macrophages stimulation with TGFβ predominantly activates the regulatory SMAD2 and −3 but activation of SMAD1 and −5 was also observed [5]. Phosphorylated SMAD2 and SMAD3 share high affinity for SMAD4 and show oligomerization, which is necessary for nuclear translocation and activation of transcription [3]. SMAD7 is an inhibitory SMAD, which negatively controls this pathway.

TGFß plays a substantial role in tumor progression, acting both as a tumor suppressor and tumor promoter [6]. Early in tumorigenesis, TGFß arrests the cell-cycle, which is SMAD-dependent. With tumor progression, tumor cells often harbor inactivating mutations in the TGFß pathway, allowing them to overcome these growth-inhibitory effects. In the tumor microenvironment, TGFß responsive cells secret cytokines and enzymes to promote EMT (epithelial mesenchymal transition), angiogenesis, tumor invasion, and metastasis. Besides, TGFß also recruits and activates immune cells [7]. Of particular interest is the induction of an alternatively activated macrophage phenotype, which promotes tumor development [8, 9]. TGFß-recruited macrophages are highly phagocytic for aberrant cells and thereby decrease the ability of tumor antigens to be delivered to the adaptive immune system [10]. Inhibition of NF-ĸB in TGFß-polarized macrophages accounts for their reduced ability to produce pro-inflammatory cytokines [7, 11].

At sites of inflammation, like in tumors, macrophages face hypoxia. Hypoxia promotes angiogenesis, fibrosis, and immune suppression [12]. Activation of the hypoxia-inducible factor (HIF) conveys many responses to hypoxia, by altering transcriptional outputs. HIF is a heterodimeric transcription factor composed of an ubiquitously expressed β-subunit and an oxygen-sensitive α-subunit [13, 14]. Three HIF-α subunits are identified in the human genome (HIF-1α, HIF-2α or EPAS1, and HIF-3α), which all share the oxygen-dependent degradation domain (ODD). In well-oxygenated tissue specific prolyl hydroxylases hydroxylate the ODD of the α-subunit, allowing their recognition by the von Hippel-Lindau tumor suppressor and subsequent proteosomal degradation [13, 14]. Under hypoxic conditions HIF-α is stabilized, translocates to the nucleus and thus, promotes transcription of target genes to induce angiogenesis, ensures cell survival, restores oxygen homeostasis but also promotes fibrosis [15]. These functions overlap with TGFß-signaling, indicating some signal crosstalk [6, 12, 16]. In renal epithelial cells and prostate cancer cells TGFß provokes HIF-1α accumulation, which in turn enhances expression of common target genes [17, 18]. In contrast, TGFß alone failed to accumulate HIF-1α in macrophages [19] even though the prolyl-hydroxylase-2 and −3 (PHD2/3) mRNA were decreased [20]. Although macrophages play an important role in fibrosis and immune suppression the impact of hypoxia on TGFß-signaling in macrophages is poorly understood. We provide evidence that TGFß-signaling under hypoxia is impaired, due to calpain-mediated SMAD2 degradation in macrophages.

## Results

### Hypoxia attenuates TGFß-induced SMAD2 activation in macrophages

**Fig. 1:**
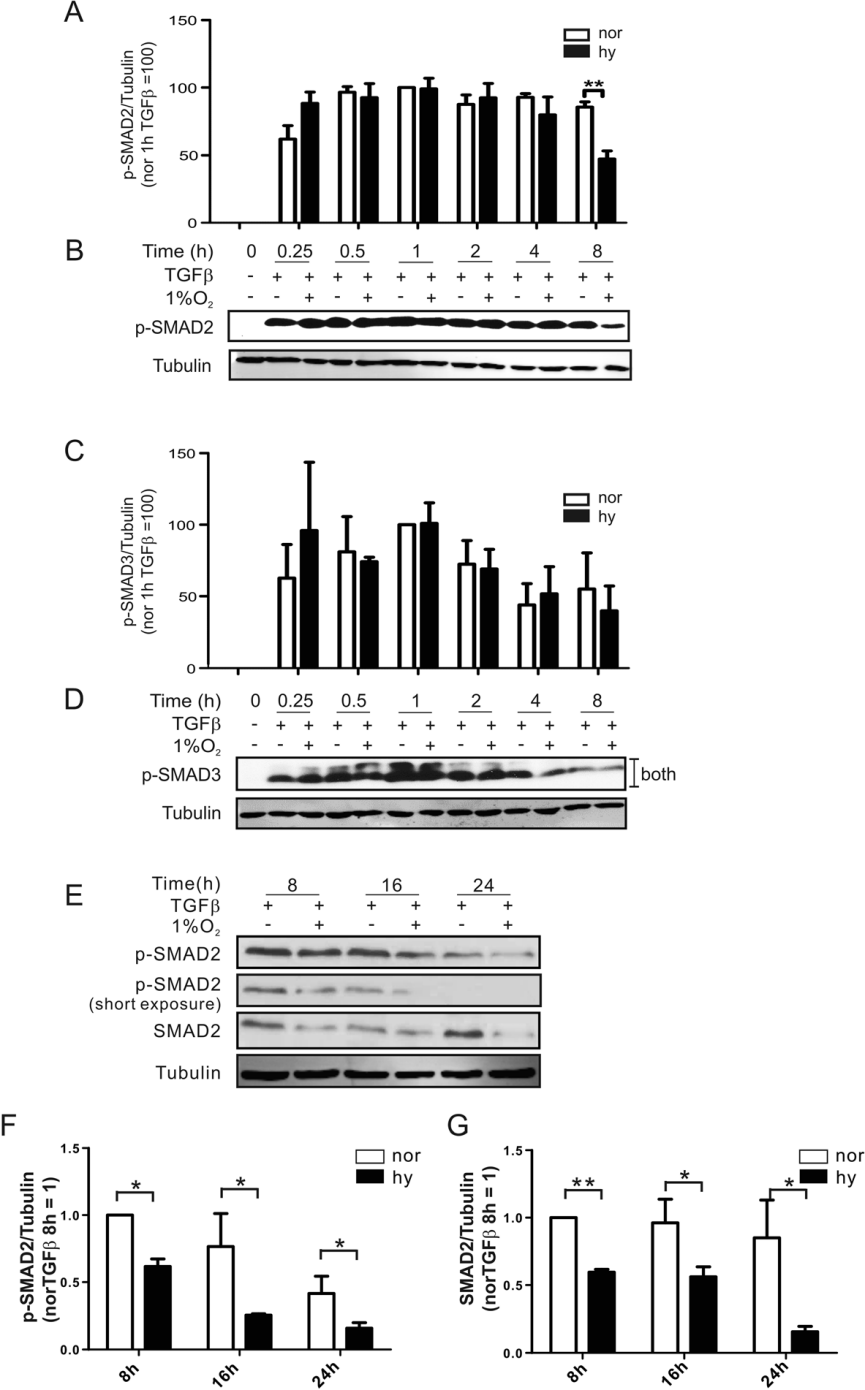
Hypoxia attenuates TGFß-induced SMAD2 phosphorylation in macrophages. Time-dependent Western blot analysis **b**, **d** and the corresponding statistical evaluation **a**, **c** of SMAD2 **a**, **b** and SMAD3 **c**, **d** phosphorylation in human primary macrophages exposed to TGFß under normoxia (−; nor) or hypoxia (1 % O_2_; hy). Macrophages were exposed to TGFß under normoxia vs. hypoxia for 8, 16, and 24 h, followed by (**e**) Western blot analysis of total SMAD2 and SMAD2 phosphorylation and corresponding statistical analysis **f**, **g**. Tubulin served as a loading control

Macrophages in the tumor microenvironment are exposed to hypoxia and TGFß but their signaling crosstalk has not been explored in detail. Therefore, we time-dependently stimulated primary human macrophages with 10 ng/mL TGFß under normoxia vs. hypoxia and followed phosphorylation of SMAD2 (p-SMAD2) and SMAD3 (p-SMAD3) by Western blot analysis. Resting macrophage did not show SMAD2 phosphorylation (Fig. 1a, b), whereas TGFß provoked a rapid SMAD2 phosphorylation within 15 min. Phospho-SMAD2 reached its maximum after 1 h both under normoxia and hypoxia. After 4 h, the level of p-SMAD2 gradually declined under hypoxia compared to normoxia. These differences became significant after 8 h (Fig. 1a, b). In contrast, the amount of p-SMAD3 rapidly increased upon TGFß addition, with phosphorylation not being affected by hypoxia. Although we noticed declining levels of p-SMAD3 at 4 to 8 h, this occurred irrespective to the presence or absence of oxygen (Fig. 1c, d). Apparently, in TGFβ-stimulated macrophages, only SMAD2, but not SMAD3, was sensitive to hypoxia. To explore this response for longer incubation times, we stimulated cells with TGFβ for 8 to 24 h at 20 % or 1 % O_2_. We observed decreased SMAD2 phosphorylation comparing 8 to 16 or 24 h, an effect drastically enhanced by hypoxia (Fig. 1e, f). Using an antibody to detect total SMAD2 protein showed a progressively reduced expression under hypoxia from 8 to 24 h (Fig. 1e, g). Apparently, hypoxia reduced phosphorylation of SMAD2 at 8 to 24 h following TGFβ-stimulation because of reduced SMAD2 protein expression.

### A decrease in SMAD2 under hypoxia is unrelated to TGFΒR2 or SMAD7

**Fig. 2:**
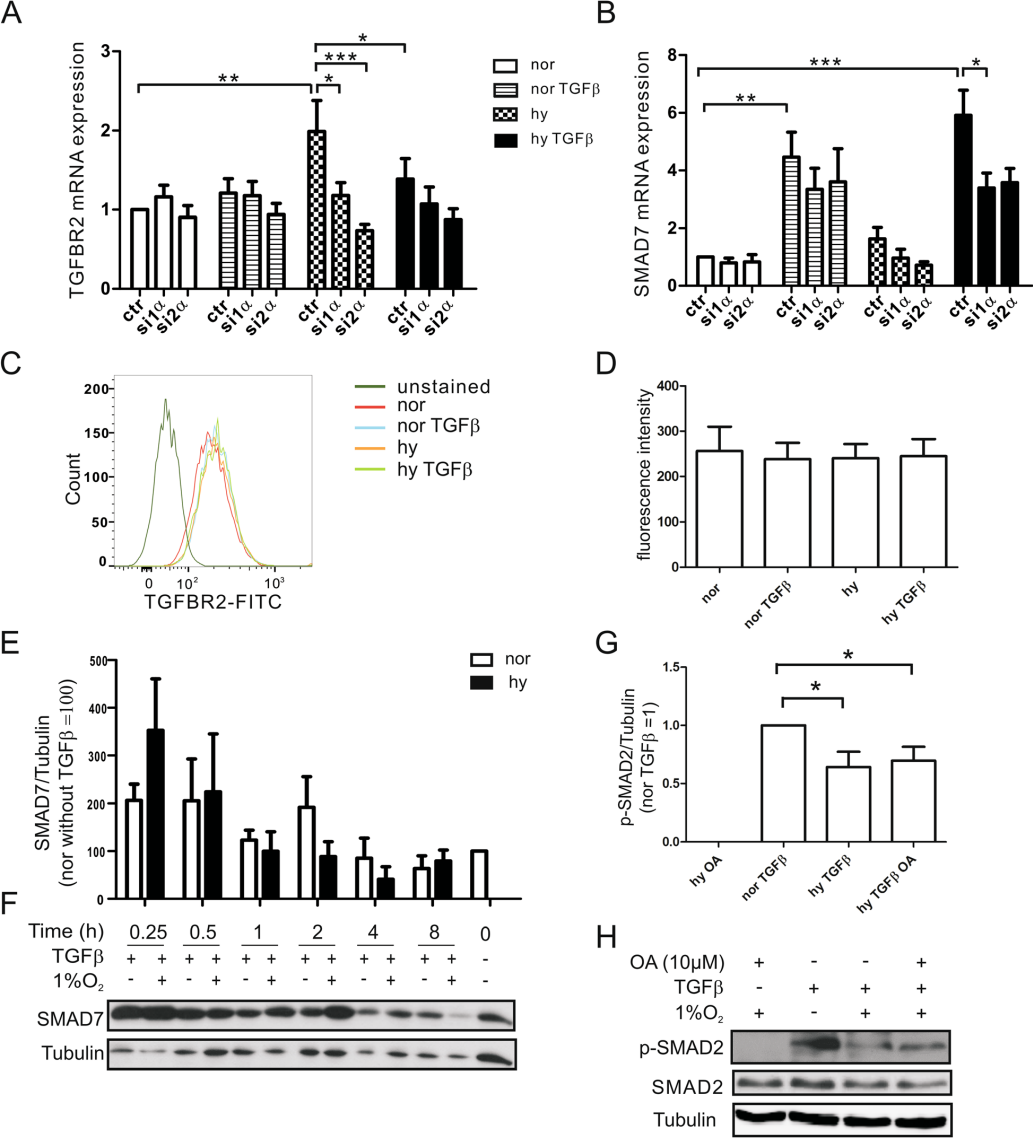
Expression of TGFBR2 and SMAD7 in response to TGFß/hypoxia. Macrophages were transfected with non-targeting siRNA constructs (ctr), siRNA-HIF1α (si1α), or siRNA-HIF2α (si2α) and mRNA expression of TGFBR2 **a** or SMAD7 **b** were analyzed by quantitative PCR after treatments with TGFß under normoxia (nor) or hypoxia (hy) for 8 h. **c** Cell surface expression of TGFBR2 was followed by flow cytometry after stimulation of macrophages with TGFß under normoxia (nor) vs. hypoxia (hy) for 8 h and **d** mean fluorescence intensity was calculated from 4 individual experiments. **e**, **f** Time-dependent Western blot analysis and statistical evaluation of SMAD7 expression in macrophages exposed to TGFß under normoxia or hypoxia. **g**, **h** Macrophages were exposed to the phosphatase inhibitor okadaic acid (OA) 1 h prior to adding TGFß for 8 h. Phosphorylation and total protein expression of SMAD2 was followed by Western blot analysis, using tubulin as a loading control

Several publications link SMAD activation to its subsequent degradation [21, 22]. Therefore, we started to validate TGFΒR2 and SMAD7 as potential HIF target genes that were previously identified by ChIP-Seq and mRNA array experiments [23]. TGFΒR2 is the receptor subunit that binds TGFß and phosphorylates the co-receptor TGFΒR1 to initiate intracellular signaling. An altered expression level of TGFBR2 will alter the ratio of TGFβ to its receptor, thereby modulating downstream signaling. SMAD7 is an inhibitory SMAD, which competes with SMAD2/3 at the TGFß receptor to control TGFß signal transmission. Increased expression under hypoxia may reduce SMAD2 activation. First, we validated microarray data by quantitative PCR 8 h after TGFß and/or hypoxia stimulation and verified hypoxic induction of TGFΒR2 mRNA (Fig. 2a). The knockdown of HIF-1α or HIF-2α, which was verified by Western blot analysis (Additional file 1: Figure S1A, B), reduced hypoxic TGFBR2 mRNA induction. mRNA expression of TGFBR2 under hypoxia/TGFß was lower compared to the hypoxic response. In contrast, SMAD7 was predominantly induced by TGFß, with the signal not being altered by a HIF-knockdown (Fig. 2b). Hypoxia only showed a small mRNA induction of SMAD7, while induction by hypoxia/TGFß was comparable to TGFß alone. A knockdown of either HIF-1α or HIF-2α slightly reduced hypoxic induction of SMAD7 and significantly reduced induction by hypoxia/TGFß, suggesting the involvement of HIF in hypoxic SMAD7 mRNA regulation.

To determine the impact of hypoxia on TGFΒR2 protein expression, we examined its surface appearance after 8 h by flow cytometry (Fig. 2c, d). TGFΒR2 expression was neither altered by TGFß nor by the combination of hypoxia/TGFß, indicating that the SMAD2 decrease is unrelated to TGFΒR2 expression. In addition, Western blot analysis verified that the expression of SMAD7 increased by TGFß at 15 to 30 min, decreased after 1–2 h and remained low up to an 8 h incubation period. However, SMAD7 protein expression was similarly affected by TGFß supplied under normoxia and hypoxia (Fig. 2e, f). Thus, it appears that neither the expression of TGFBR2 nor of SMAD7 account for the specific effects of hypoxia seen at the level of SMAD2.

**Fig. 3:**
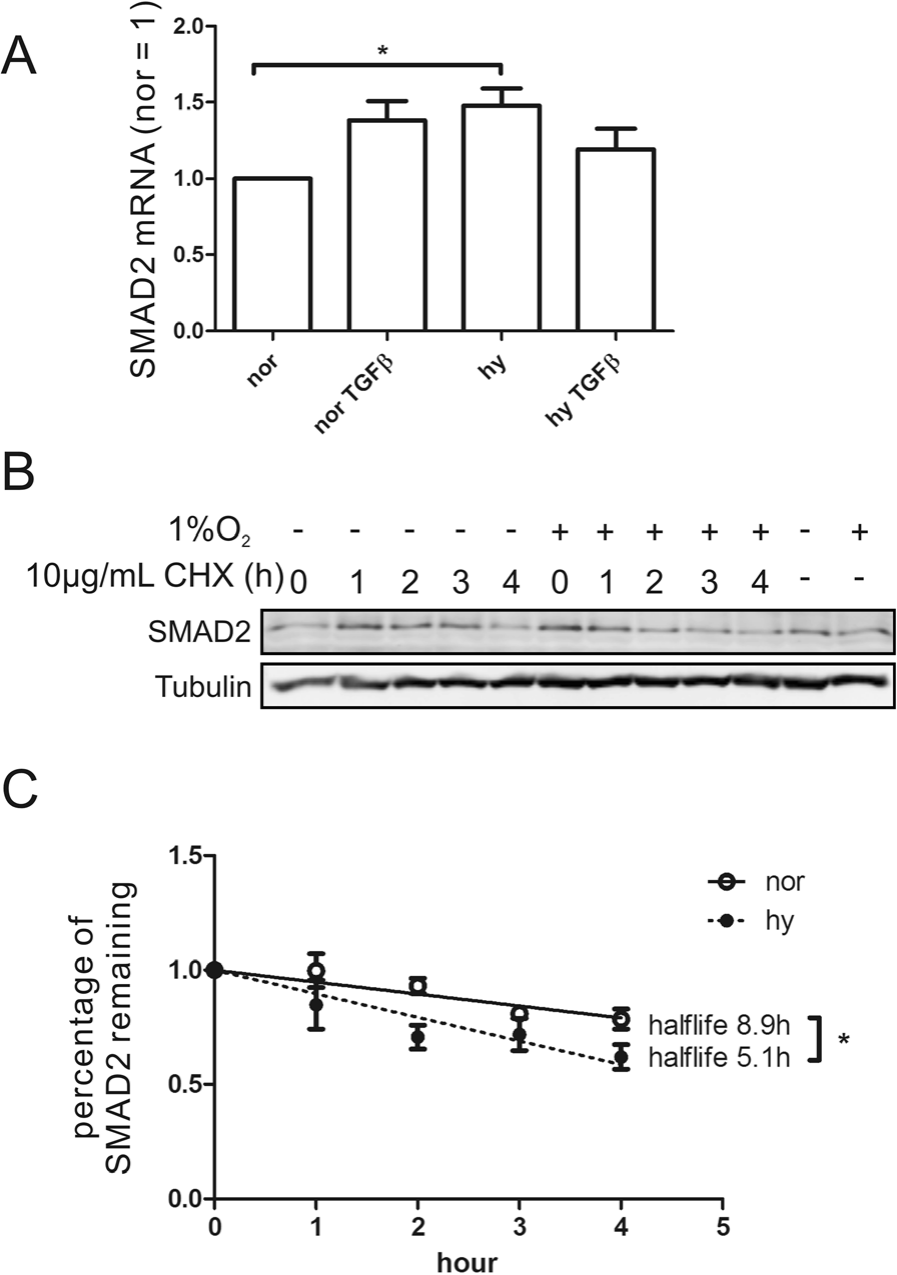
SMAD2 protein stability is reduced in response to TGFß/hypoxia. **a** mRNA expression of SMAD2 followed by quantitative PCR in macrophages stimulated for 8 h with TGFß under normoxia (nor) vs. hypoxia (hy). **b** Macrophages were stimulated with TGFß for 4 h, followed by the addition of cycloheximide for 0 h to 4 h and subsequent Western blot analysis of SMAD2 expression. **c** Half-life determination of SMAD2 in TGFß-stimulated macrophages under normoxia (nor) vs. hypoxia (hy) as a result of experiments performed in Fig. 3b

Next, we considered phosphatases to decrease SMAD2 phosphorylation. It was reported that PP2A dephosphorylates p-SMAD3 under hypoxia [24]. Supplying the PP2A inhibitor okadaic acid (OA) 1 h prior to addition of TGFß for 8 h failed to rescue the decrease of phoshorylated or total SAMD2 under TGFß/hypoxia (Fig. 2h). Excluding SMAD2 activation or dephoshorylation as an explanation for its decreased protein amount, we went on to analyze SMAD2 mRNA. In general, protein expression is either regulated by an altered mRNA expression, reduced translation, and/or enhanced degradation. SMAD2 mRNA expression was slightly induced but not reduced by hypoxia (Fig. 3a). Therefore, we examined protein stability by suppressing translation using 10 μg/ml cycloheximide (CHX). SMAD2 was degraded faster, comparing hypoxia to normoxia (Fig. 3b). The half-life of SAMD2 under normoxia/TGFß was calculated to be 8.9 ± 1.2 h, while it was reduced to 5.1 ± 0.6 h under hypoxia/TGFß (Fig. 3c). In conclusion, SMAD2 is destabilized under hypoxia in TGFß-stimulated macrophages.

### SMAD2 degradation under hypoxia is facilitated by calpain

**Fig. 4:**
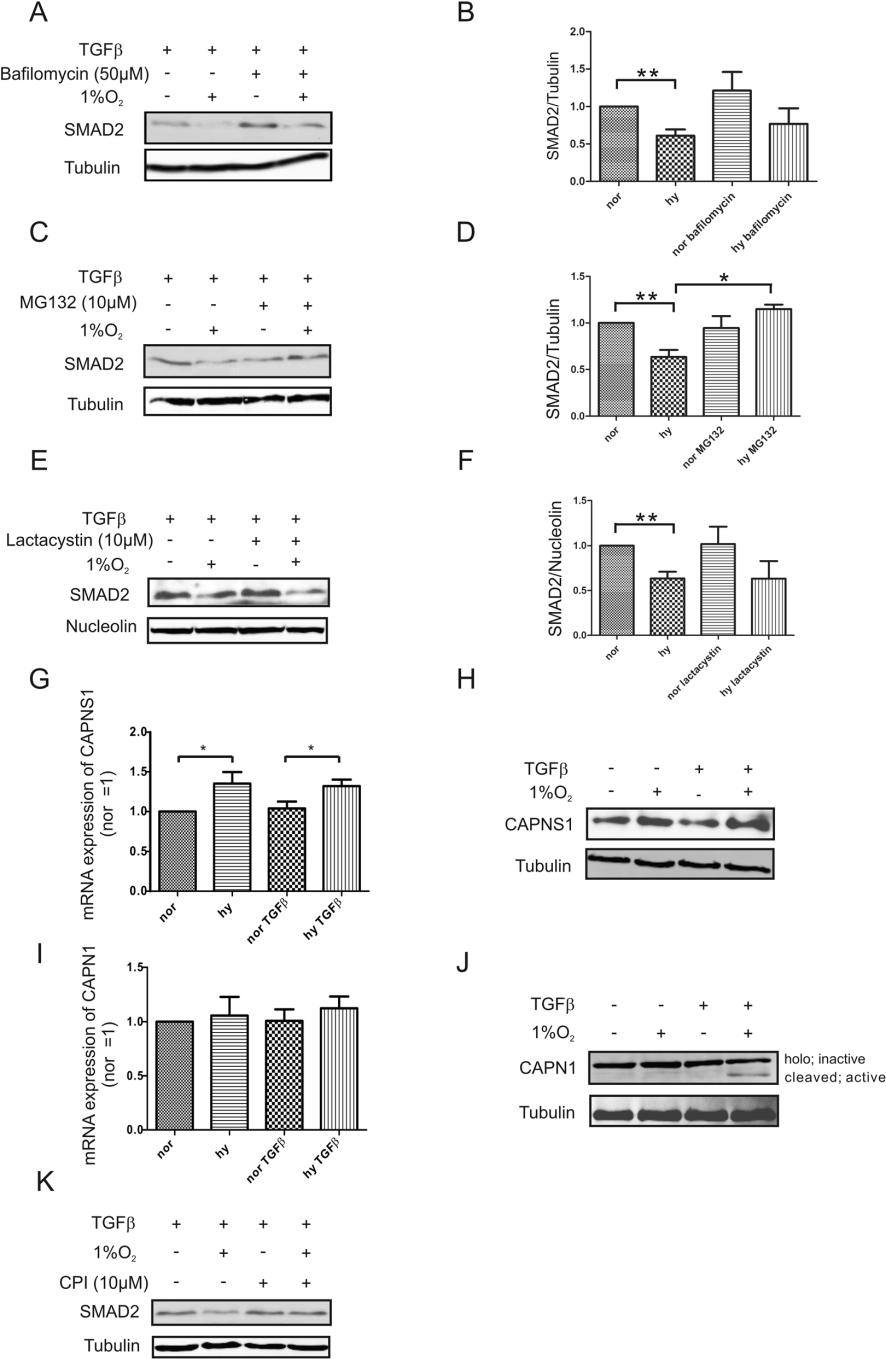
SMAD2 degradation under hypoxia is facilitated by calpain. **a**-**f** Macrophages were stimulated with TGFß under normoxia vs. hypoxia for 8 h in the presence of bafilomycin **a**, **b**, MG132 **c**, **d**, or lactacystin **e**, **f**. 2 h after TGFß, inhibitors were added and incubations went on for 6 h. Western blot analysis and statistical evaluation is presented. **g** mRNA expression of the calpain regulatory subunit (CAPNS1) was analyzed by quantitative PCR in macrophages exposed for 8 h to TGFß under normoxia (nor) vs. hypoxia (hy). **h** Western blot analysis of the calpain regulatory subunit after exposure to TGFß and/or hypoxia for 8 h. **i** mRNA expression of the calpain 1 catalytic subunit (CAPN1) was analyzed by quantitative PCR in macrophages exposed for 8 h to TGFß under normoxia (nor) vs. hypoxia (hy). **j** Proteolytic cleavage of CAPN1 under TGFß/hypoxia was followed in macrophages by Western blot analysis after 8 h. **k** Macrophages were exposed to the calpain inhibitor 1 (CPI) to follow SMAD2 degradation after stimulating cells for 8 h with TGFß under normoxia vs. hypoxia

Considering increased degradation of SMAD2 under TGFß/hypoxia, we analyzed pathways being involved. Using bafilomycin A1 to inhibit lysosomal functions, SMAD2 degradation remained unaffected thus, ruling the involvement of the lysosomal compartment out (Fig. 4a, b). We then used MG132 to block proteasomal- and calpain-dependent degradation systems. MG132 rescued SMAD2 degradation in response to TGFß/hypoxia, while lactacystin, a more specific proteasomal inhibitor, not affecting calpain, failed to restore SMAD2 expression under these conditions (Fig. 4c-f). Calpain is a calcium-dependent non-lysosomal cysteine proteolytic system that comprises a small regulatory subunit (CAPNS1, also known as calpain reg) and a large catalytic subunit (μ-calpain/m-calpain, also known as CAPN). We tested mRNA expression of CAPNS1 in macrophages stimulated with TGFß under normoxia and hypoxia. CAPNS1 mRNA was significantly upregulated by hypoxia or hypoxia/TGFß compared to normoxia or TGFß-stimulation (Fig. 4g). The mRNA increase became also apparent at the protein level (Fig. 4h). Expression of the catalytic subunit (CAPN1) was not affected by TGFβ or hypoxia nor their combination (Fig. 4i). Following macrophage activation with hypoxia/TGFß, holo-calpain (80KD) is proteolytically processed and the removal of 14 or 28 amino acids forms active calpain. Western blot analysis detected active calpain in macrophages stimulated with TGFß/hypoxia but not with TGFβ or hypoxia alone (Fig. 4j). Experiments with the specific calpain inhibitor 1 CPI (10 μM), showed that decreased SMAD2 expression seen under hypoxia/TGFß was fully recovered when calpain activity was blocked (Fig. 4k). Conclusively, in TGFß-stimulated macrophages hypoxia promotes degradation of SMAD2 by increasing calpain activity.

**Fig. 5:**
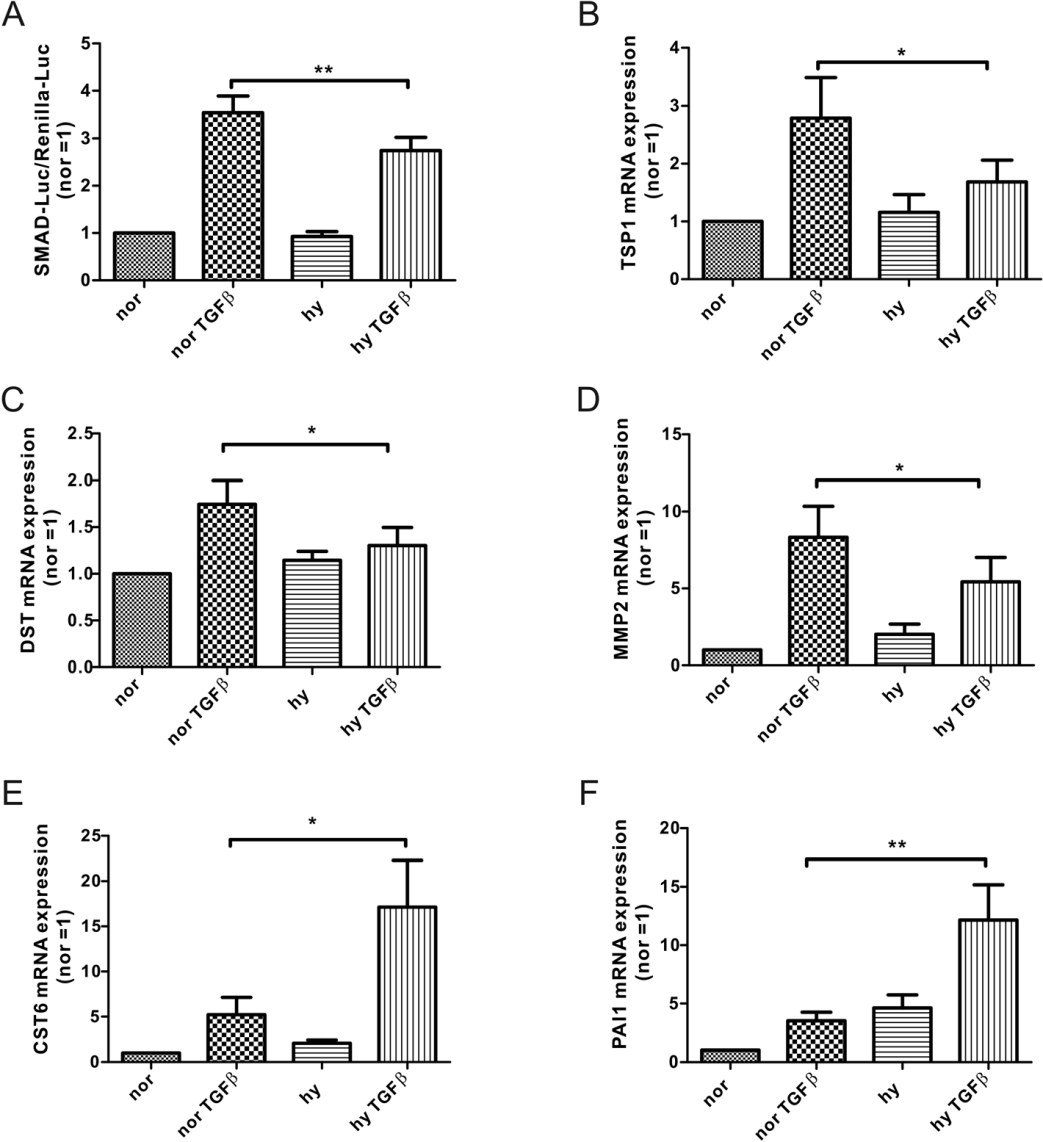
Hypoxia reduces SMAD2-dependent transcription. **a** TGFß-induced transcriptional activity in J774A.1 cells transfected with a SBE4-Luc (4xSMAD binding element coupled to luciferase) as well as a Renilla-Luc plasmid. Stimulation was with TGFß under normoxia (nor) vs. hypoxia (hy) for 8 h. mRNA expression of **b** thrombospondin1 (TSP1), **c** dystonin DST, **d** matrix metalloproteinase (MMP2), **e** cystatin (CST6), and (**f**) plasminogen activator inhibitor 1 (PAI1) was analyzed by quantitative PCR in macrophages exposed for 8 h to TGFß under normoxia (nor) vs. hypoxia (hy)

To explore how hypoxia affects TGFß-induced transcriptional activity, we used a SMAD-specific reporter assay. Due to difficulties in transfecting primary human macrophages we used the J774A.1 mouse macrophage cell line, which responded with SMAD2 phosphorylation in response to TGFß, also showing a decrease in SMAD2 expression with TGFß/hypoxia under longer exposure times (Additional file 1: Figure S1C, D). Stimulation of J774A.1 cells, transfected with the SMAD reporter plasmid SEB4-Luc, with TGFß under hypoxia significantly reduced transcriptional activity compared to TGFß supplied under normoxia (Fig. 5a). We then analyzed expression of SAMD2 and SMAD3 target genes in primary human macrophages. Thrombospondin 1 (TSP1), dystonin (DST), and matrix metalloproteinase 2 (MMP2) were reported to be selectively regulated by SMAD2 [25–27] and we confirmed their responsiveness to TGFβ (Fig. 5b, c, d). Induction was significantly reduced in macrophages stimulated with TGFβ at 1 % O_2_, reflecting the reduced expression of SMAD2. In contrast induction of SMAD3 target genes such as cystatin (CST6) and plasminogen activator inhibitor 1 (PAI1) [25] was not reduced under hypoxia (Fig. 5e, f). Apparently, only transcriptional responses of SMAD2 are selectively reduced by hypoxia in TGFβ-stimulated human macrophages.

## Discussion

In human macrophages, in contrast to epithelial and parenchymal cells [17, 18], periods of 8 h hypoxia or longer reduced TGFß-induced SMAD2 activation. Decreased phosphorylation of SMAD3, but not SMAD2, under hypoxia was noticed in HeLa cells and attributed to protein phosphatase 2A (PP2A) directly interacting with SMAD3 [24]. In macrophages the decrease of p-SMAD2 was not due to PP2A activation, altered TGFBR2 receptor activation, or inhibitory SMAD7 expression, but rather resulted from hypoxia-evoked destabilization of SMAD2. Regulation of SMAD2 degradation is complex. Smurf2 is an E3 ubiquitin ligase, directly interacting with activated SMAD2 and promoting its proteasomal degradation. Neural precursor cell expressed developmentally down-regulated protein 4–2 (NEDD4-2), another E3 ubiquitin ligase like Smurf1 or 2, binds SMAD2 and SMAD3 but exclusively degrades SMAD2 [21]. In addition, TGFβ-induced factor homeobox 1 (TGIF) interacting ubiquitin ligase 1 (Tiul1), induces SMAD2 to bind TGIF, which triggers SMAD2 degradation [22]. Our data add proteolysis by the calpain system also to account for alterations in the SMAD2 protein amount. Interestingly, Lo and coworkers studied the proteasomal degradation of SMAD2 [28]. Along our observations, their results suggested that lactacystin cannot rescue a SMAD2 decrease to the same extent as MG132, although they did not consider the calpain system.

The calpain system consists of a catalytic subunit (μ-calpain/m-calpain) and a common regulatory subnunit CAPNS1 as well as the inhibitory subunit calpastatin. After calcium binding, calpastatin detaches from the protein complex and calpain is activated by autolysis [29]. The calpain system plays important roles in macrophages. IFNγ induces calpain mRNA and protein expression in U937 and THP1 cells [30]. Calpain cleaves iNOS and thus, affects inflammatory responses [31]. There are several reports that calpain activity is upregulated under hypoxia, with platelets being one typical example [32–35]. Hypoxia-induced thrombogenesis is associated with CAPNS1-dependent calpain activation in the platelet activation cascade [35]. In line, we noticed increased calpain activity under TGFβ/hypoxia by upregulating CAPNS1 in macrophages. The calpain regulatory subunit functions like a chaperone, assisting the catalytic subunit to fold properly [36]. We showed that combining TGFß and hypoxia is necessary to activate calpain in human macrophages as indicated in Fig. 4j, while hypoxia alone did not activate calpain. This is in line with findings showing that ischemia/hypoxia provokes an influx of Ca^2+^ into the cell [32, 35], but the Ca^2+^ increase is not sufficient to activate calpain [29, 37]. It is well established that TGFß could also increase the influx of Ca^2+^ into cells [38]. Presumably, a high expression of CAPNS1 and the influx of Ca^2+^ by the combined stimulation, hypoxia plus TGFß, promotes activation of calpain. Calpain digests proteins at a limited number of cleavage sites producing large polypeptide fragments rather than small peptides or amino acid [29]. Early studies suggested that preferred residues of the cleavage P_2_ position are Leu, Thr, and Val, and in the P_1_ position are Lys, Thr, and Arg [39]. Following the P_2_-P_1_ rule, SMAD2 has 17 potential cleavage sites. In addition, calpain prefers small hydrophilic Ser residue at the P_1_’ position. Considering this preference, there are still 4 possible cleavage sites at K46, Y101, R108, Y266 for calpain in SMAD2. On the other hand, there are indications that the specificity for calpain is not exclusively governed by amino acid but rather by the 3D conformation of the substrates [29, 39]. Using currently available online tools at http://calpain.org for calpain substrates, amino acids at sites 166, 395, and 6 are the top 3 predicted cleavage sites with a score of 0.39, 0.39, 0.37 respectively [40]. For the identification of the accurate cleavage sites more experiments are needed that would be beyond the scope of this work.

Our experimental conditions aimed to simulate conditions macrophages are facing when entering the tumor microenvironment, facing hypoxia and TGFß [41]. SMAD2 and SMAD3 have distinct transcriptional roles in the tumor [27, 42, 43]. SMAD2 provokes secretion of anti-angiogenetic factors, such as thrombospondin 1 (TSP1, Fig. 5) as well as the VEGF-A antagonist sFlt-1, whereas SMAD3 induces the secretion of pro-angiogenetic factor, i.e., VEGF-A or plasminogen activator inhibitor 1 (PAI1, Fig. 5), promoting angiogenesis when SMAD2 is inactive [26, 44]. In addition, TSP1 overexpression in tumor cells enhances the recruitment of pro-inflammatory macrophages and reduces tumor growth [45]. Conditional medium of SMAD2 knockdown fibroblast induces proliferation and *in vivo* deletion of SMAD2 in tumor cells generates a more aggressive phenotype compared to controls [26]. The decrease of SMAD2 under TGFβ/hypoxia in tumor-associated macrophages may add to their roles supporting angiogenesis and tumor progression.

Angiogenesis influences tumor growth but also enables metastasis [46]. Metastasis is a complex process requiring invasive growth of tumor cells, epithelial to mesenchymal transition (EMT), induction of angiogenesis, intra- and extravasation, and tumor growth at the secondary sites [47]. Matrix metalloproteases (MMPs) like MMP2 coordinate multiple steps of this process. In general they promote metastasis by allowing extracellular matrix remodeling and releasing growth factors like VEGF from the matrix. MMP2 was also implicated in endothelial cell apoptosis [46, 48]. As full activation of MMP2 by the membrane type (MT)1-MMP provokes endothelial cell death and expression of MMP2 in endothelial cells and macrophages is reduced in acute hypoxia (Fig. 5d), one might speculate that this reduction prevents endothelial cell death under hypoxia when angiogenesis is usually enhanced [48, 49]. Other TGFß-induced genes involved in metastasis are enhanced under hypoxia since SMAD3 target genes like PAI-1 or CST6 (Fig. 5) are regulated by the hypoxia-inducible factor (HIF) as well [16, 50]. In a previous study we used ChIP-seq technology to identify HIF-1 and HIF-2 binding sites in primary human macrophages and identified, amongst others, CST6 and PAI-1 [23]. Apparently, in these cases TGFß/hypoxia enhances rather than decreases gene expression in macrophages. Thus, specifically attenuating SMAD2 under TGFß/hypoxia might have functional roles on macrophages biology and tumor progression.

## Conclusion

**Fig. 6:**
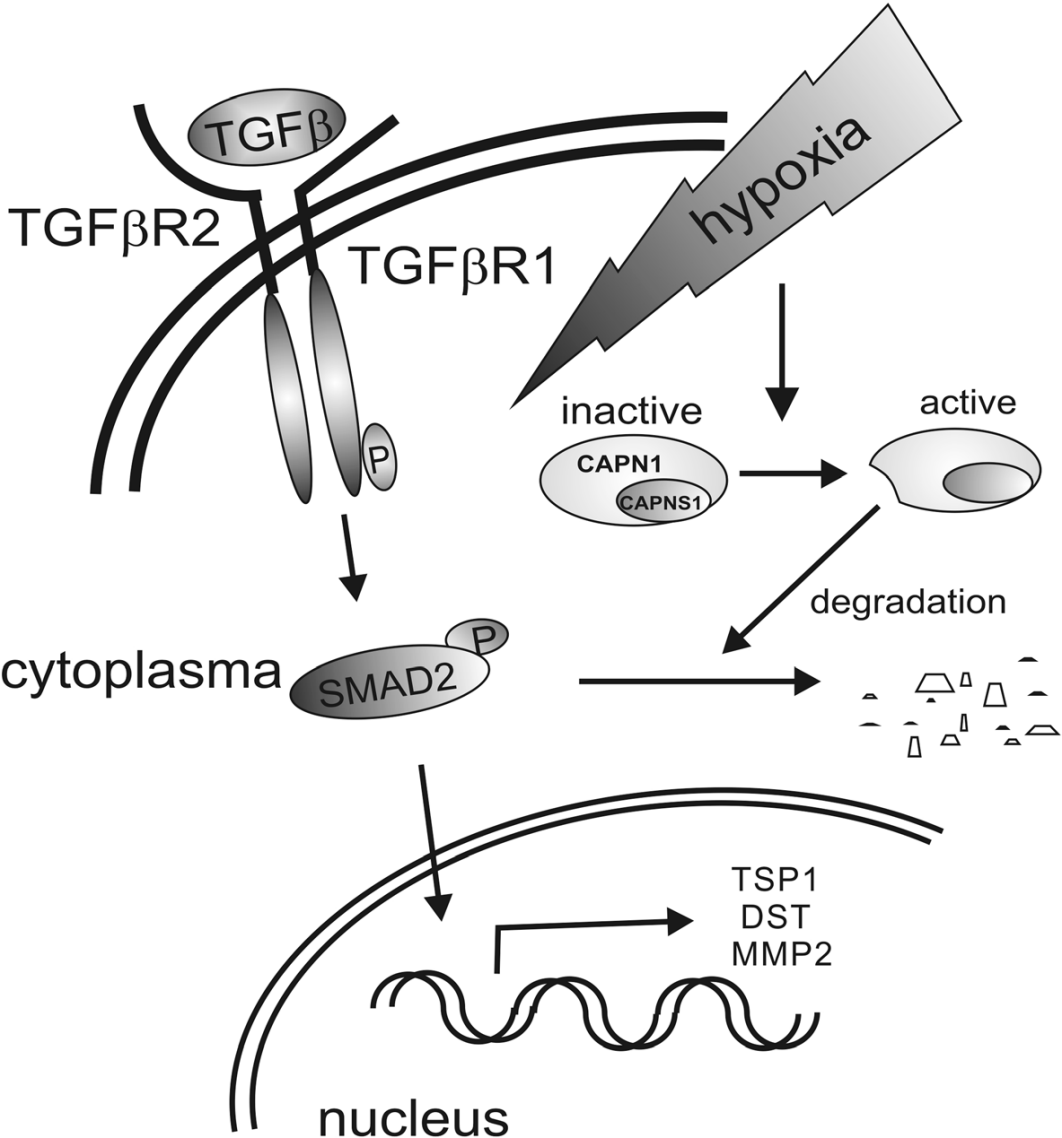
Graphical abstract. TGFβ binds to its receptor (TGFβR1 and TGFβR2), induces phosphorylation of TGFβR1 and downstream signaling molecules like SMAD2. Active SMAD2 translocates to the nucleus to provoke expression of target genes, i.e., TSP1, DST, and MMP2. Hypoxia in combination with TGFβ activates calpain to degrade SMAD2, which in turn attenuates SMAD2-dependent transcriptional activation and thus, diminishes the induction of TSP1, DST, and MMP2

Our results indicate that hypoxia attenuates SMAD2 activation in TGFβ-stimulated macrophages (Fig. 6). Mechanistically, this results from degradation of SMAD2 by calpain.

## Material and methods

### Cell culture and incubation procedures

If not indicated otherwise, all chemicals were purchased from Sigma (Steinheim, Germany), while cell lines came from ATCC (LGC Promochem, Wesel, Germany). J774A.1 cells were cultured in Dulbecco’s Modified Eagle Medium with 10 % fetal calf serum, 100 U/ml penicillin, and 100 μg/ml streptomycin (PAA Laboratories, Cölbe, Germany). Primary human monocytes were isolated from 50 ml buffy coats (DRK Blutspendedienst Baden-Württemberg-Hessen, Frankfurt, Germany). Blood was layered on a Ficoll-Isopaque gradient (P = 1077 g ml^−1^). The interphase containing peripheral blood mononuclear cells was obtained after centrifugation (800 × g, 20 min). Cells were recovered, washed twice in PBS, and left to adhere on culture dishes (Sarstedt, Nümbrecht, Germany) for 90 min at 37 °C. Non-adherent cells were removed. The medium was changed to fresh RPMI 1640 medium, supplemented with 3 % (v/v) human plasma (HP), 2 mM L-glutamine, 100 U/ml penicillin, and 100 μg/ml streptomycin (PAA Laboratories, Cölbe, Germany). Cells were kept at 37 °C in a humidified atmosphere with 5 % CO_2_. Monocytes were differentiated with human plasma for 6–8 days before they were treated with 10 ng/ml TGFß. Purity of macrophage cultures was about 90 %. To establish hypoxic conditions cells were incubated in a hypoxic workstation with 1 % O_2_, 94 % N_2_, 5 % CO_2_ (Invivo2 400, Ruskinn Technology, Leeds U.K.). Protein stability was assessed by stimulating macrophage with TGFß for 4 h, followed by 10 μg/mL cycloheximide (CHX). Samples were harvested after additional 1, 2, 3, or 4 h. Degradation of SMAD2 was followed by the addition of 10 μM lactacystin, 10 μM MG132 (Z-Leu-Leu-Leu-al), or 50 μM bafilomycin A1. After 2 h incubations with TGFß co-incubations with TGFß and inhibitors went on for 6 h. Phosphatase PP2A was inhibited by 10 μM okadaic acid (OA), preincubated for 1 h. 10 μM calpain inhibitor 1 was added simultaneously together with TGFß to macrophages.

### Transfection of primary human macrophages

For siRNA transfections human macrophages were seeded in 6-well plates as described above. 50 nM siRNA against HIF-1α (ON-TARGETplus SMART pool, Human HIF1A, Thermo Scientific, Karlsruhe, Germany), HIF-2α (ON-TARGETplus SMART pool, Human EPAS1, Thermo Scientific), or non-targeting siRNA pool (GE Healthcare Dharmacon) was used with 16.8 μl HiPerfect (Qiagen, Hilden, Germany) in 500 μl medium with penicillin and streptomycin for each well. After adding the mix, cells were incubated for 24 h. Then medium was removed and replaced by medium containing penicillin, streptomycin and human serum to start experiments.

### Western blot analysis

Cells were lysed in lysis-buffer (6.65 M urea, 10 % glycerol, 1 % SDS, 10 mM Tris/HCl pH 6.8, pH was adjusted to 7.4) and sonicated. After centrifugation (16.000 × g, 10 min) the protein content in the supernatants was determined by a protein assay kit (Bio-Rad, Munich, Germany), 70 μg total protein was separated on 10 % SDS gels and blotted on nitrocellulose membranes. The following antibodies were used: human pSMAD2 (#3108, Cell Signaling Technology), human pSMAD3 (ab52903, Abcam), human SMAD7 (ab124890 Abcam), human SMAD2/3 (#3102 Cell Signaling Technology), human calpain regulatory subunit (sc-32785, Santa Cruz), CAPN1 (C5736, Sigma), nucleolin (sc-13057, Santa Cruz), and ß-tubulin (T4026, Sigma). Horseradish peroxidase-conjugated goat anti-rabbit or anti-mouse IgG (Sigma) were used as secondary antibodies. Densitometry was performed using image J software (http://rsbweb.nih.gov/ij/). Briefly, rectangles were used to assess signal strength of each individual band and the background was measured by using the baseline tool and subtracted from individual values. Target protein expression was normalized to the loading control i.e., tubulin or nucleolin.

### Luciferase reporter activity

The SBE4-Luc reporter plasmid was a gift from Bert Vogelstein (Addgene plasmid # 16495) [51], while the renilla control vector pRL-SV40 was bought from Promega (Frankfurt, Germany). J774A.1 cells were transiently transfected with 0.2 μg plasmid using JetPrime™ transfection reagent (Polyplus transfection, Illkirch, France) according to the manufacturer's protocol. After 16 h, medium was changed and cells were starved for 2 h prior to stimulation. Afterwards, cells were lysed and firefly as well as renilla luciferase activities were determined using a Dual Luciferase kit assay (Promega) on a Mithras LB 940 luminometer (Berthold, Bad Wildbad, Germany).

### Flow cytometry (FACS)

For TGFΒR2 surface expression, single cell suspensions were generated from detached macrophages by digestion with accutase (PAA) for 10 min at 37 °C. 10^5^ cells were transferred to new tubes. Before staining, non-specific antibody binding to Fcγ receptors was blocked with Fcγ Block Receptor Binding Inhibitor (eBioscience, Frankfurt, Germany) for 15 min on ice, followed by staining with human TGFΒR2 fluorescein-conjugated antibody (FAB241F,R&D Systems, Germany) using standard protocols. We used an LSRFortessa Cell Analyzer (BD, US) and results were analyzed using FlowJo software 7.6.1 (Treestar, Ashland, OR, USA). To discriminate macrophages from other non-leukocyte cells, samples were stained with CD45 (BD Bioscience). For daily instrument calibration we used Cytometer Setup and Tracking beads (BD Biosciences).

### mRNA isolation and quantitative PCR

Isolation of mRNA and quantitative PCR was performed as described in [52]. Primers are:

- SMAD2, forward: CCGACACACCGAGATCCTAAC,
- reverse: GAGGTGGCGTTTCTGGAATATAA;
- SMAD7, forward: CCAGGCTCCAGAAGAAGTTG,
- reverse: CCAACTGCAGACTGTCCAGA;
- TGFΒR2, forward: GGAAACTTGACTGCACCGTT,
- reverse: CTGCACATCGTCCTGTGG;
- CAPNS1, forward: GACACCCTGATCTGAAGACTGA,
- reverse: GCCTGCCACCTTTTGATGTT;
- CAPN1, forward: ACATGGAGGCCATCACTTTC
- reverse: GGTCCACGTTGTTCCACTCT
- TSP1, forward: CCAGATCAGGCAGACACAGA
- reverse: AGTTGTCCCGTTCATTGAGG.
- DST, forward: GATGCAGATCCGAAAACCCCT
- reverse: CTCAGTGCGGTCCAGTTGTA
- MMP2, forward: AGGGCACATCCTATGACAGC
- reverse: ATTTGTTGCCCAGGAAAGTG
- CST6, forward: CTACTTCCGAGACACGCACA
- reverse: GGAACCACAAGGACCTCAAA
- PAI1, forward: AGCTCCTTGTACAGATGCCG
- reverse: ACAACAGGAGGAGAAACCCA
- TBP, forward: GGGCCGCCGGCTGTTTAACT,
- reverse: AGCCCTGAGCGTAAGGTGGCA.

### Statistics

Experiments for mRNA expression and flow cytometry were performed in duplicate and repeated at least 3 times. Western blot analysis was performed at least 3 times. Data are expressed as means ± SEM. Multigroup statistically significant differences were calculated after analysis of one-way variance (ANOVA) and Bonferroni’s test. A statistically significant difference between two groups was calculated by paired *T* test. Individual *P* values are given in the figures (*P < 0.05, **P < 0.01, ***P < 0.001).

## Additional file

Additional file 1: Figure S1. Western blot analysis of (A) HIF-1α and (B) HIF-2α of primary human macrophages transfected with non-targeted siRNA constructs (ctr), siRNA-HIF1α (si1α), or siRNA-HIF2α (si2α) exposed to normoxia vs. hypoxia (1% O_2_) for 8 h. (C) Western analysis of phospho SMAD2 in J774 cells, exposed to TGFß under normoxia vs. hypoxia (1% O_2_) for 8 h. (D) Statistical analysis of data presented in Figure S1C.
