# Supplementary material for: Hypoxia induces calpain activity and degrades SMAD2 to attenuate TGFβ signaling in macrophages
Source: Cell Biosci. 2015 Jul 4;5:36. doi: 10.1186/s13578-015-0026-x (PMC4491253; doi:10.1186/s13578-015-0026-x)
Supplement: Additional file 1: Figure S1. — Western blot analysis of (A) HIF-1α and (B) HIF-2α of primary human macrophages transfected with non-targeted siRNA constructs (ctr), siRNA-HIF1α (si1α), or siRNA-HIF2α (si2α) exposed to normoxia vs. hypoxia (1% O2) for 8 h. (C) Western analysis of phospho SMAD2 in J774 cells, exposed to TGFß under normoxia vs. hypoxia (1% O2) for 8 h. (D) Statistical analysis of data presented in Figure S1C. [file 13578_2015_26_MOESM1_ESM.docx]

**Additional file**

**
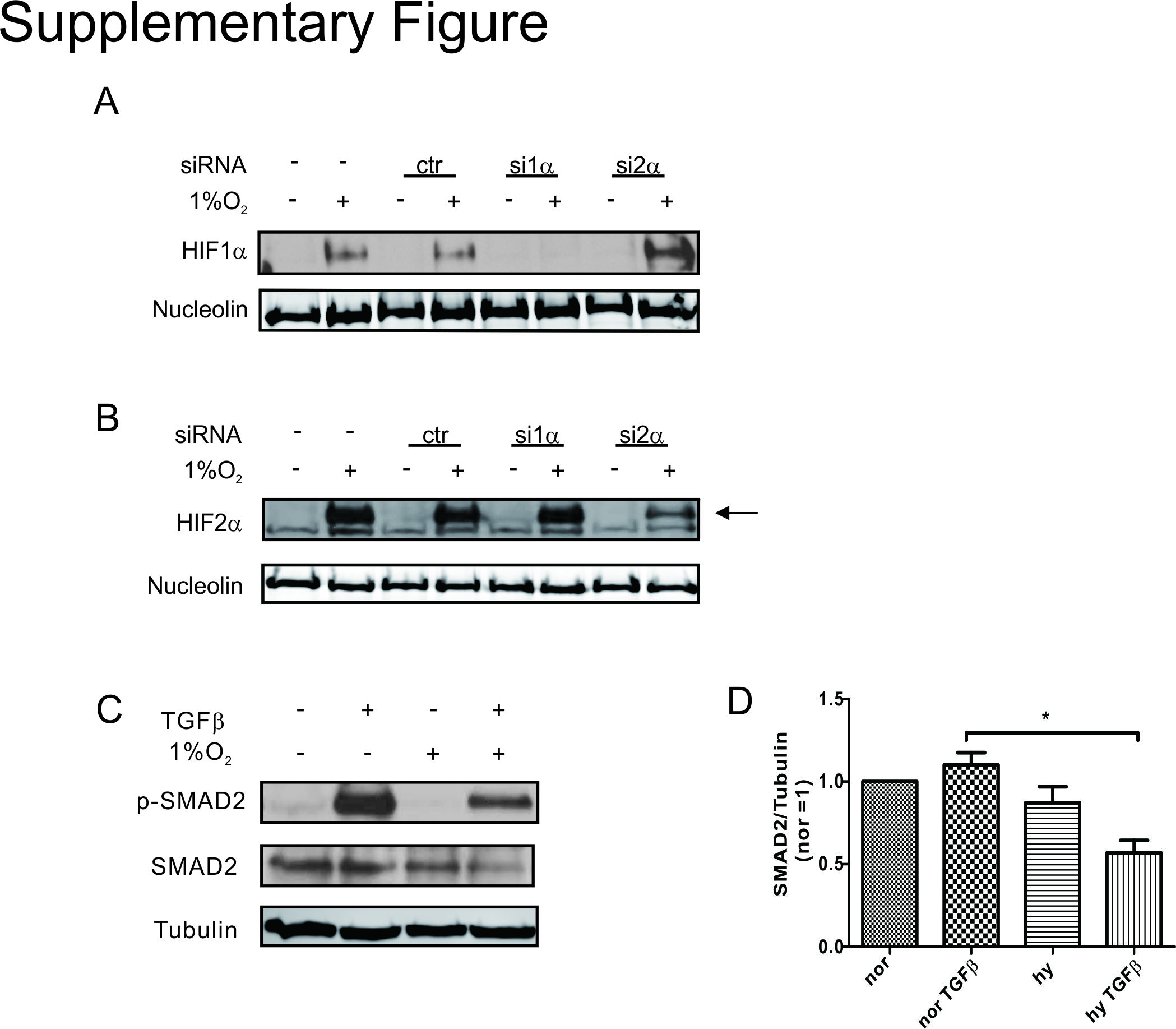
**

**Additional file 1: Figure S1.**

Western blot analysis of (A) HIF-1α and (B) HIF-2α of primary human macrophages transfected with non-targeted siRNA constructs (ctr), siRNA-HIF1α (si1α), or siRNA-HIF2α (si2α) exposed to normoxia vs. hypoxia (1% O_2_) for 8 h.

(C) Western analysis of phospho SMAD2 in J774 cells, exposed to TGFß under normoxia vs. hypoxia (1% O_2_) for 8 h. (D) Statistical analysis of data presented in Figure S1C.
